# Measuring Physical Neighborhood Quality Related to Health

**DOI:** 10.3390/bs5020190

**Published:** 2015-04-29

**Authors:** Kimberly A. Rollings, Nancy M. Wells, Gary W. Evans

**Affiliations:** 1 School of Architecture, University of Notre Dame, 110 Bond Hall, Notre Dame, IN 46556, USA; 2 Design and Environmental Analysis, Cornell University, 1411 Martha Van Rensselaer Hall, Cornell University, Ithaca, NY 14853, USA; E-Mail: nmw2@cornell.edu; 3 Design and Environmental Analysis, Human Development, and the Bronfenbrenner Center for Translational Research, Cornell University, 1411 Martha Van Rensselaer Hall, Cornell University, Ithaca, NY 14853, USA; E-Mail: gwe1@cornell.edu

**Keywords:** physical neighborhood environment, health, measurement, causality, spatial scale

## Abstract

Although sociodemographic factors are one aspect of understanding the effects of neighborhood environments on health, equating neighborhood quality with socioeconomic status ignores the important role of physical neighborhood attributes. Prior work on neighborhood environments and health has relied primarily on level of socioeconomic disadvantage as the indicator of neighborhood quality without attention to physical neighborhood quality. A small but increasing number of studies have assessed neighborhood physical characteristics. Findings generally indicate that there is an association between living in deprived neighborhoods and poor health outcomes, but rigorous evidence linking specific physical neighborhood attributes to particular health outcomes is lacking. This paper discusses the methodological challenges and limitations of measuring physical neighborhood environments relevant to health and concludes with proposed directions for future work.

## 1. Introduction

Although sociodemographic factors are one aspect of understanding the effects of neighborhood environments on health, equating neighborhood quality (NQ) with socioeconomic status (SES) ignores the important role of neighborhood physical attributes. Physical NQ attributes potentially relevant to health include: land use, density, street connectivity, transportation availability and infrastructure, pedestrian and cycling infrastructure (presence, condition, and maintenance of sidewalks, bike lanes, cross walks, street lights, traffic lights); access to nature and green space, public and open spaces, and resources (public services, health care, healthy food, schools, playgrounds, commercial functions, and recreational opportunities); building and street condition, cleanliness, and maintenance; and traffic volume, air quality, and noise. Research documents the growing recognition of physical NQ effects on morbidity; obesity and related chronic health outcomes, such as greater diabetes and hypertension; health behaviors, such as diet and physical activity; mental health outcomes, such as stress, anxiety, and depression; and social well-being outcomes, including social interaction, social cohesion, and social capital (see reviews: [1,2,3,4,5,6,7,8,9,10,11,12,13,14,15,16,17,18,19,20,21,22,23,24,25]). Although many studies have focused on isolated NQ attributes, such as green space [26], and characteristics immediately surrounding a residence rather than a defined spatial neighborhood area [27], few studies have measured overall physical NQ using reliable and validated measurement tools [4]. This paper summarizes the methodological challenges and limitations of measuring physical NQ within the context of health. We conclude by outlining directions for future work.

## 2. Methodological Challenges and Limitations of Measuring Physical NQ

### 2.1. Research Design and Causal Inference

Most research on physical NQ and health relies on cross-sectional studies. Variability in both physical NQ and individual residents within a cross-sectional sample affects the study’s internal validity; sufficient variability is necessary for analysis [28]. Although understanding neighborhood health effects in particular contexts and among particular populations is useful (see Moderators and Mediators), cross-sectional results must not be interpreted to suggest that physical attributes have no effect on health; the lack of significant results may be attributed to truncated variance. For example, sampling only rural neighborhoods may exclude neighborhood attributes relevant to health such as traffic, noise, and air pollution that are typically present in more urban areas. The distribution of health effects must also be understood when sampling individual residents in order to achieve appropriate variability. Effects of physical NQ on individual resident health likely vary by age. Children, for instance, may spend more time at home than adolescents and, thus, exposure to neighborhood characteristics relevant to health increases with age. More rigorous methodologies such as natural experiments, quasi-experiments, and longitudinal studies are needed to establish causal relations [19,29]. Moving to Opportunity (MTO) public housing voucher program provides a rare natural experiment—randomly assigning public housing tenants to voucher programs enabling them to move to different types of neighborhoods or remain in public housing—but did not directly evaluate a neighborhood-level intervention; results could not differentiate between effects of housing changes from effects of physical NQ changes on health. Rigorous study of neighborhood health effects throughout the life course, as neighborhoods change, and as residents move further requires tracking both individuals and neighborhoods over time [30].

#### 2.1.1. Selection Bias

Selection bias renders causal inference difficult in many NQ studies. Typically, neighborhood residents select themselves into residential environments based on individual traits potentially related to health outcomes [1,5]. Sociocultural and economic factors, in addition to preferences related to, for example, diet, transportation, and physical activity, affect neighborhood selection. Individuals who enjoy being physically active may choose to live in communities designed to promote walking, such as those guided by principles of New Urbanism. Health-conscious residents who eat well, regularly exercise, and experience little stress may additionally choose to live in what they perceive as a healthy neighborhood [31]. Similarly, low SES people tend to live in deprived neighborhoods and experience additional stressors that contribute to health outcomes compared to people of moderate or high SES [32,33]. Thus, it is problematic to attribute variance in health outcomes to NQ [28]. For example, low-SES neighborhoods often have higher prevalence of depression [34]. Individual SES, however, is strongly positively linked to depression [1,28]. More rigorous research methods such as prospective studies and natural experiments are needed to address selection bias. Selection bias is typically addressed by adjusting for individual-level confounds, such as SES, via inclusion of covariates in regression or multilevel models, and then estimating associations between neighborhoods and health [1]. However, this approach to dealing with bias presumes that one has adequate measures of all the most plausible confounding variables.

#### 2.1.2. Time

Neighborhood conditions are dynamic and can change in a short period of time (e.g., presence of disorder, noise, *etc.*) [35]. Therefore, the timing and frequency of neighborhood observations are critical. Time of day (e.g., morning *vs.* evening), type of day (weekday *vs.* weekend or holiday), and season (e.g., winter *vs.* summer) must be considered when conducting observations of neighborhoods and health outcomes. Additionally, the location from which the observations are conducted may also be important. Walking or driving down the street *versus* sitting inside and observing through a window could yield different results [3]. Furthermore, neighborhood effects on health may vary over time as a function of age, require time to accumulate, or may not be observed until after some period of time or lag [1]. Relevant timing of observations, similar to spatial scale, may differ based on particular health outcomes and neighborhood context. For example, effects of neighborhood conditions on obesity or BMI may require exposure to physical NQ that affects health behaviors for a long period of time. However, effects of physical NQ on physical activity may be observed more quickly. Children rarely present chronic disease morbidity and, thus, early markers of risk are more sensitive (e.g., blood pressure *versus* cardiovascular disease). Examining cumulative and lagged neighborhood health effects is challenging and requires longitudinal tracking of neighborhood changes and residents, as well as identifying available data for multiple regions as residents move to multiple neighborhoods over time [36].

#### 2.1.3. Neighborhood Boundaries and Spatial Scale

Spatial definition of neighborhoods is challenging and has been operationalized in multiple ways [1,4,37,38]. *Administrative boundaries* (e.g., U.S. Census geographies) are often used because complete data are freely available for many conterminous areas [1]. The correlation between administrative and neighborhood boundaries, however, as well as the relevance of these boundaries in neighborhoods and health studies, is inconsistent [1,4,38]. Although census tracts vary in size, their typically large area often requires smaller areas to be sampled for research purposes [6]. GIS methods and software facilitate measurement of physical NQ within *smaller areas surrounding individual locations* via street network or “crow-fly” distances, such as a quarter-mile or 10-min walking distance in studies of walkability. Forsyth and colleagues [39] examined built environments by using buffers of diverse spatial scales and zones. Results related to diet and physical activity significantly differed by spatial measure. Spatially defining neighborhoods by areas that are conceptually too large or small for a hypothesized neighborhood-health relation can bias estimates of neighborhood effects [38,40]. Also known as the modifiable areal unit problem, the type of geographic boundary used to aggregate data can affect variance, standard deviations, correlation, and regression analyses [38]. Correlations are often—but not always—more significant for larger geographic units, overestimating neighborhood effects on health [38]. Unless boundaries of neighborhood factors relevant to health coincide with administrative boundaries, underestimation of neighborhood effects on health can occur due to exposure misclassification [41]. Use of larger administrative neighborhood definitions can bias estimates of exposure especially for measures of proximity; larger differences between measurements of distance to nearest facilities from address-based *versus* census-based boundaries can result from samples containing more residents residing near census-unit boundaries rather than distributed within the boundary [40]. Perceived, *resident-defined neighborhood boundaries* may better indicate actual access and exposure to destinations, resources, and walking routes than administrative data [42]. Resident-defined boundaries, however, may be affected by perceptions of neighborhood reputation; residents might report living in positively-perceived neighborhoods but exclude themselves from stigmatized areas [38]. Appropriate neighborhood definition may vary widely by size, location, context, and question depending upon the health outcome of interest [1]. Defining neighborhood boundaries requires that researchers carefully consider factors relevant to specific health outcomes in addition to legal jurisdictions, place names, architectural character consistency, and administrative geographies [43]. Until theories are developed suggesting associations between spatial scales and particular health outcomes, studies should conduct exploratory analyses to test results at various spatial scales [1].

#### 2.1.4. Moderators and Mediators

Identifying moderating factors that alter the relation between physical NQ and health is important both for sensitive analyses and for informing policy and practice. Items in neighborhood assessment instruments relevant to health must consider theories appropriate for particular community and cultural contexts. Potential moderators to be examined when estimating effects of physical NQ on health include sociodemographics, such as age, gender, SES, and culture; contextual factors, such as urbanity; psychosocial variables, such as crime; and social environment attributes, such as social cohesion [44]. Different physical characteristics of neighborhoods are likely more relevant to some health outcomes than others. Proximity to food retail establishments with fresh produce, for instance, is likely associated more directly with diet and physical health than social well-being. For example, some evidence suggests that effects of proximity to healthy food may be exacerbated among low SES populations [45]. The introduction of a supermarket into a neighborhood without any markets improved consumption of healthy foods, but only for those who previously had poor dietary habits [46]. In the MTO study, female but not male youth who relocated from inner city, public housing projects to middle SES neighborhoods experienced improvements in mental health relative to their peers remaining in low SES neighborhoods [47].

An additional challenge in estimating physical NQ effects on health is distinguishing between confounding factors and underlying mechanisms (mediators). Pedestrian infrastructure, for instance, can influence physical activity which in turn affects physical health measures such as BMI or blood pressure. Examinations of mediators via more complex statistical methods, such as structural equation modeling, that account for mediators, moderators, and correlates of health will contribute to understanding the mechanisms through which physical NQ affects health. Studying specific neighborhood attributes also raises methodological challenges related to isolating individual attribute effects on health when many neighborhood properties are related [30]. Some attributes of the physical environment are inseparable from social factors such as subjective perceptions and make isolating independent effects difficult [2]. For example, perceptions of crime and safety likely relate to availability and use of pedestrian infrastructure and outdoor physical activity facilities when evaluating outdoor physical activity. Furthermore, areas outside of neighborhoods where residents spend time may also need to be considered when examining pathways of neighborhood effects on health, such as around schools or workplaces [48]. Recognizing the multilevel nature of neighborhoods and health research is critical, as neighborhoods are located within larger jurisdictions that can exert macro forces on health [41].

### 2.2. Measuring Physical Neighborhood Attributes

#### 2.2.1. Subjective Measures

The use of perceived measures of neighborhood has advantages and disadvantages. Directly communicating with neighborhood residents can reveal information that is not apparent during direct observations (such as presence of trash or other disorder), and document actual use of and exposure to various neighborhood facilities and conditions [3,28]. If neighborhood residents regularly spend most of their time in locations outside of the defined neighborhood boundary, resulting effects of neighborhood conditions on health may be over- or underestimated. Resident reports also provide data about the experience of neighborhood conditions, which can vary by individual [28]. Despite the advantages of subjective neighborhood resident reports, several disadvantages threaten their validity. Residents may incorrectly perceive distances or inaccurately identify attributes within neighborhood boundaries [17]. Furthermore, perceptual biases and individual differences can affect neighborhood ratings especially when health outcome data are also self-reported, resulting in same-source or monomethod bias [49]. When neighborhood conditions and health outcomes are both subjectively reported by residents, variance in both measures is related and amplifies the correlation between neighborhood attributes and health outcomes. Measurement error in both neighborhood and health measures is also correlated, and health outcomes could affect subjective perceptions, potentially yielding spurious results. Physically active neighborhood residents, for example, may be more likely than less active neighbors to report available facilities. Resident self-reports of neighborhood attributes may be less varied than objective measures; the lack of variability can generate insignificant results suggesting that certain physical NQ characteristics contribute little to explanations of between-neighborhood variations in health outcomes [50].

#### 2.2.2. Census-Derived and Indirect Measures

Indirect, objective measures of neighborhood include aggregate proxy measures such as those calculated from administrative (e.g., census) data; and other secondary spatial data such as street network and land-use data analyzed using GIS methods and software. Despite the high availability and feasibility of administrative (census) data, data collection frequency, geographic scale, and availability of social and housing data that describe the built environment are limited [51]. Census data measure socioeconomic variables to estimate neighborhood composition, but exclude contextual neighborhood data on infrastructure, presence of institutions, resources, facilities and levels of cleanliness and maintenance [50,52]. Analyses using aggregated proxy data misestimate neighborhood effects on health due to errors inherent in using, for example, neighborhood SES as a proxy measure for physical NQ [1,28,53,54,55,56]. Specific physical NQ attributes also cannot be identified by analyses using aggregated proxy measures.

The recent increase in use of GIS methods and software has improved measures of physical NQ including proximity, density, land use and accessibility of resources, and factors associated with urban form such as street connectivity and networks, land use, and population and residential density [11]. GIS software allows for examining fixed distances using circular or street network “zones” around individual neighborhood residences, accounting for more variability within larger areas and assisting in reconciling data sets collected within different sized administrative or geographic areas. Because data are often not current or verified for accuracy by researchers, accuracy and validity of results can be limited [17]. Studies that focus on the area immediately surrounding a residence, however, fail to consider effects of features and resources available in more distant areas on health [1,51]. Low-SES neighborhoods, for example, that are spatially isolated from resources may suffer more negative effects on health than deprived areas closer to resource-rich neighborhoods [1]. Measures using GIS procedures may also fail to account for quality of and actual access to amenities related to health outcomes [22]. Access to a basketball court, for instance, may be more important than overall counts of physical activity facilities within defined neighborhood boundaries [57]. Parents and children may visit neighborhoods that are not necessarily nearby their residence to access facilities, potentially due to social networks, transportation availability, or perceptions of crime and safety. Fear of crime, as well as disamenities, such as traffic volume, may interfere with access to proximate destinations. Not accounting for quality and access could result in misestimation of health effects of the physical environment, again highlighting the need for theory and studies examining spatial scales relevant to specific health effects [1,58,59,60,61].

*Aerial photography and satellite imagery* used with GIS procedures indicate presence and quantity of physical NQ features, but not actual use, access, or quality [17]. Neighborhood attributes visible via imagery may be inaccessible financially (payment required for use), physically (fence or locked gate; must cross a busy street to access), or legally (hours of operation or privately owned facility requiring membership). Furthermore, *other secondary data sources* (e.g., municipal records, telephone directories) have also been used to map and measure specific attributes, such as food retail establishments and playgrounds, available within certain areas [46,62]. Although more appropriate than aggregated indices of deprivation, the data have not been verified and assume that listed facilities are in fact present and operational [17]. Reliance on aggregated, census-derived and secondary data alone yields an oversimplified analysis of neighborhood effects on health and often neglects within-neighborhood variability [63].

#### 2.2.3. Objective, Direct Measures

In order to address limitations of subjective and secondary or aggregated proxy measures of neighborhood attributes, detailed objective direct and systematic social observations (SSO) by trained outside raters have been used to study neighborhood attributes [35,64]. SSO researchers directly observe selected blocks of interest intended to represent overall neighborhood area to assess factors such as cleanliness and maintenance, disorder and incivilities, and presence of facilities, public space, and natural elements [3]. Direct neighborhood measures avoid same-source bias and tautological reasoning when both neighborhood and health outcomes are self-reported [28]. Direct measures can further capture neighborhood conditions that are not perceived by residents that also affect their health [65]. Observation scheduling, however, must account for relevant times of day, week or weekend day, and season [3]. Otherwise, only attributes that are visible and readily identifiable during times of observation can be documented [3].

Despite their benefits, direct measures present multiple challenges. Although direct measures can provide a more accurate account of existing neighborhood conditions, they are more labor-intensive, time consuming, and expensive than indirect measures due to training, travel to neighborhood sites, and data collection, especially for large scale, longitudinal studies. Moreover, aggregating observational data to the neighborhood level assumes that observed blocks are representative of the overall neighborhood [3]. Selecting appropriate areas or streets to directly observe can be challenging as neighborhood features can sometimes vary widely even within small areas. Furthermore, validated direct, objective neighborhood measures also require evaluation and appropriate levels of both inter-rater and test-retest reliability. Inter-rater reliability assesses the consistency of neighborhood ratings between observers. Achieving appropriate levels of reliability and internal validity, especially for qualitative assessment items (ratings of maintenance, disorder) when compared to quantitative attributes (presence, counts, proximity), requires resources and time to train multiple observers. Direct measures can also place observers at risk in unsafe neighborhoods, and raise, ethical concerns when documenting neighborhood conditions via photographs or videos without resident permission [3,4].

Although agreement between subjective and objective neighborhood measures has been moderate to low [66,67,68], both are needed in studies of neighborhood effects on health. When compared to objective measures, subjective measures of neighborhood attributes describe different constructs [22]. Perceptions of physical NQ assess items from residents’ point-of-view, at ground-level. Self-reported data about physical NQ may be influenced by other factors, such as perceived access, crime, and safety [69]. Subjective ratings of safety, crime, and behavior can result in more significant predictions than objective measures [41]. Objective measures of physical NQ, such as quantities or proximity of nearby amenities and resources indicate little beyond the number and proximity of potentially available resources present in a defined area and might not distinguish between settings that actually encourage positive health outcomes and settings that are uninviting, inaccessible, or unused [70,71]. No information regarding access to and actual use of facilities is gathered (e.g., safety, operating hours, affordability, operational status). Additionally, no distinction is made between facilities that do and do not actually promote positive health outcomes. Objective measures of physical NQ potentially address more quantitative measures while subjective measures reflect more qualitative measures of neighborhood environments [70,71].

## 3. Future Directions

Given the complexity of physical NQ and health relations, a combination of strategies will be required to address the challenges and limitations of physical NQ and health research. First, development and application of theories linking physical NQ—as well as spatial boundaries and scales—to specific health outcomes are needed. Many frameworks used in existing NQ and health work stem from other fields, such as sociology, and were not intended for use in studies of health outcomes. Second, multiple and more rigorous methodologies, including natural and quasi-experiments; rigorous observational, longitudinal, and simulation studies; advanced multilevel and structural equation modeling analyses; and both objective and perceived physical NQ measures will assist with addressing challenges, such as self-selection, lack of cross-study comparison, and better examine causal direction. Furthermore, qualitative methodologies may suggest relevant hypotheses to test via larger quantitative data sets, and assist with interpretation of quantitative study results [30]. Third, development and testing of physical NQ indices relevant to specific health outcomes, temporal dynamics, and spatial scales are also necessary. A previous review identified few studies that examined psychometric properties of observational measures [3]. Fourth, identifying moderating factors—where, when, and for whom various physical neighborhood attributes affect health—can highlight areas for and inform NQ interventions. Additionally, exploring pathways (*i.e.*, mediators) by which physical NQ affects health is critical not only for study validity, but also informing policy. Rather than only identifying associations, studies must aim to explain *how* physical NQ relates to health via mediating variables. Finally, understanding effects of specifically physical NQ on health is necessary to inform design professionals aiming to improve human health. Collaborations between researchers, communities, practitioners, and policy makers, in addition to a variety of methodological strategies, will be required to inform effective interventions, practice, and policy.
